# Systematic Analysis of Drug Targets Confirms Expression in Disease-Relevant Tissues

**DOI:** 10.1038/srep36205

**Published:** 2016-11-08

**Authors:** Vinod Kumar, Philippe Sanseau, Daniel F. Simola, Mark R. Hurle, Pankaj Agarwal

**Affiliations:** 1 Computational Biology, GlaxoSmithKline, 709 Swedeland Road, King of Prussia, PA 19406, USA; 2 Computational Biology, GlaxoSmithKline, Gunnels Wood Road, Stevenage, SG1 2NY, UK

## Abstract

It is commonly assumed that drug targets are expressed in tissues relevant to their indicated diseases, even under normal conditions. While multiple anecdotal cases support this hypothesis, a comprehensive study has not been performed to verify it. We conducted a systematic analysis to assess gene and protein expression for all targets of marketed and phase III drugs across a diverse collection of normal human tissues. For 87% of gene-disease pairs, the target is expressed in a disease-affected tissue under healthy conditions. This result validates the importance of confirming expression of a novel drug target in an appropriate tissue for each disease indication and strengthens previous findings showing that targets of efficacious drugs should be expressed in relevant tissues under normal conditions. Further characterization of the remaining 13% of gene-disease pairs revealed that most genes are expressed in a different tissue linked to another disease. Our analysis demonstrates the value of extensive tissue specific expression resources.both in terms of tissue and cell diversity as well as techniques used to measure gene expression.

Tissue specificity is an important aspect of many diseases that reflects the potentially different roles of proteins and pathways in diverse cell lineages^1^. Although a variety of diseases have tissue specific etiology, many diseases ultimately affect multiple organs and tissues^2^. Genes with tissue-specific patterns of expression and function play key roles in the physiological processes of complex organisms, and such genes are regarded to be intrinsic components of many human diseases^2^. In particular, gene activity has been reported to vary more greatly across organs or tissues within an individual than in the same tissue across individuals^3^.

Large scale genome-wide analysis of gene expression patterns has routinely been used to study human disease, as it enables the comprehensive comparison of different tissues^4^,^5^,^6^. To gain insight into the genes, pathways, and mechanisms affected by disease, most studies utilizing this approach have focused on comparing disease and non-disease states^4^,^5^,^6^. However, understanding a gene’s normal pattern of expression in different healthy tissues provides a meaningful complementary perspective as well. For example, Lage *et al*.^7^ presented the first quantitative study of the tissue-specific mRNA expression of over 2,000 Mendelian disease-associated genes and showed these genes are selectively expressed only in tissues where their disruption causes pathology—even under non-disease conditions. This finding supports an important mechanistic hypothesis that pathogenic gene dysregulation tends to be localized to the tissue(s) in which the affected genes are already expressed.

In our present study, we focused on addressing a specific prediction of this hypothesis, that disease-associated genes targeted by marketed and Phase III drugs (i.e., those genes that have clinical evidence relating them to diseases and thus also a high-quality subset of disease-modifying genes) are in particular expressed in the appropriate disease-relevant tissue even under normal/non-disease conditions. The rationale for this study is to evaluate whether confirming expression of a novel drug target in the appropriate disease-relevant tissue should serve as an early and perhaps necessary step when selecting that gene to pursue for a particular disease indication. To perform this evaluation, we integrated both tissue specific mRNA and protein expression data to overcome the inherent technical limitations of each individual source.

## Results

**Figure 1:**
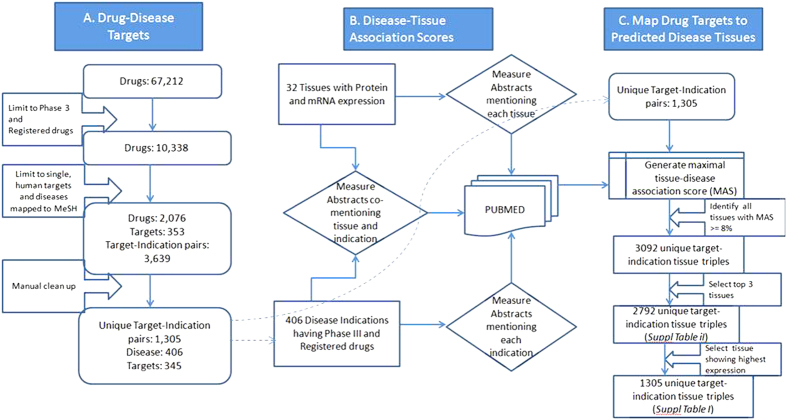
Work flow with the key filtering and processing steps applied to generate the final set of drug-target–tissue combinations investigated in this study. (**A**) Gold standard set of gene-disease relationships obtained by extracting all genes and their disease indications that are currently in Phase III or marketed as per the drug industry pipeline. (**B**) Generate a MAS score to assess the connection between a particular disease and tissue. (**C**) Mapping the relevant tissues to each drug-disease pair.

We investigated if drug targets are expressed in disease-relevant tissues under normal conditions. For the purpose of this analysis, disease-relevant tissue means healthy samples from specific tissues that are biologically relevant to the disease; it does not imply samples from patients who have the disease. The targets considered here are limited to proteins encoded by human genes. An overview of the data sources, filtering and processing applied is provided in Fig. 1. We compiled a gold standard set of gene-disease relationships by extracting the genes and their disease indications for all drugs that are currently in Phase III or marketed as per the drug industry pipeline database Pharmaprojects (http://www.citeline.com/products/pharmaprojects/) as of July 2014. This produced a set of 1,305 unique gene-disease pairs spanning 345 target genes and 406 diseases (Fig. 1A). Tissue assignments for each disease are based on the maximal association score (MAS) threshold (Fig. 1B), following the protocol developed by Lage *et al*.^7^ (see Methods). We then determined whether a given gene was expressed in a disease-associated tissue by computing a binary tissue-specific expression profile from 32 healthy tissues based on the RNA-Seq data generated by Uhlen *et al*.^8^. Figure 1C illustrates the workflow for assigning relevant tissues to each drug targets based on their expression patterns in normal healthy tissues.

Based on this assignment of tissue-specific expression to gene-disease pairs, a total of 1,081 (83%) gene-disease pairs showed detectable expression in one or more of the predicted tissue assignments (see Methods). When we further restricted each gene-disease association to the single tissue with the highest MAS score, the number of genes expressed in the assigned tissue was reduced to 969 (74%). These observations are comparable to the findings from a previous study reporting that 71% of 920 gene-disease pairs compiled from literature sources are expressed in a disease-relevant tissue^9^. To determine if the observed expression levels for these genes are elevated specifically in the disease-relevant tissues, we averaged (across all gene-disease pairs) their normalized expression (*z-*scores, see Methods) and compared them to average expression in all remaining tissues. The average *z* -normalized expression level for the targets is over three-fold higher (0.57 vs 0.16; fpkm: 79.6 vs 23.7) in the disease-relevant tissues compared to remaining tissues (P = 1.6 e^−12^; paired t-test). These results indicate that not only is the mRNA of a typical drug target expressed in the healthy version of the disease-relevant tissue, it is normally expressed at a significantly higher level in this healthy tissue compared to other tissues. Thus, broad panels of tissue-specific mRNA expression data provide valuable information when evaluating drug targets.

To assess if successful drug targets are also expressed at the protein-level, we constructed tissue-specific profiles using protein data obtained from the Human Protein Atlas (HPA)^8^. These HPA data were generated using an antibody-based detection method that indicates the spatial distribution of a given protein at the single-cell level in the various substructures and cell types of a tissue. In contrast to RNA-seq data, these protein measurements are qualitative and are labeled as ordinal variables to describe relative abundance (i.e., Absent, Low, Medium and High). In our analysis, we further summarized these values into two states, where Absent and Low become “Undetected”, while Medium and High become “Expressed”. For consistency with the binary protein expression profiles, we converted the RNA-seq data into binary values, where genes showing mRNA expression levels over 1 FPKM were classified as “Expressed” and the remaining are labeled as “Undetected”. Using these estimates, we identified 737 gene-disease pairs showing detectable protein expression. Unsurprisingly, this is lower than the 1,081 associations detected at the mRNA level, which can be attributed to the significantly better detection limit (reduced false negative rate) provided by current RNA-Seq technologies^10^.

Despite the identification of fewer expressed genes using protein expression data, some genes may be detected at the protein but not mRNA level, due to non-linear amplification during translation. Hence, we integrated both mRNA and protein expression data sets to obtain a more comprehensive estimate of the total number of gene-disease pairs with detectable expression levels in disease-relevant tissue. In this way, we identified 1,137 (87%) pairs with detected expression in the assigned tissues at either mRNA or protein levels (Supplementary Table S1). A majority (681, or 60%) of these gene-disease pairs are detected at both mRNA and protein levels. However, 400 gene-disease pairs were detected exclusively at the mRNA level and an additional 56 pairs only at the protein level (Supplementary Table S2). Table 1 shows a selected subset of the unique gene-disease pairs and their predicted tissue assignments. The entire list for all 1,305 unique gene-disease pairs spanning 345 targets and 406 diseases with their tissue assignments can be found as Supplementary Table S2.

**Figure 2:**
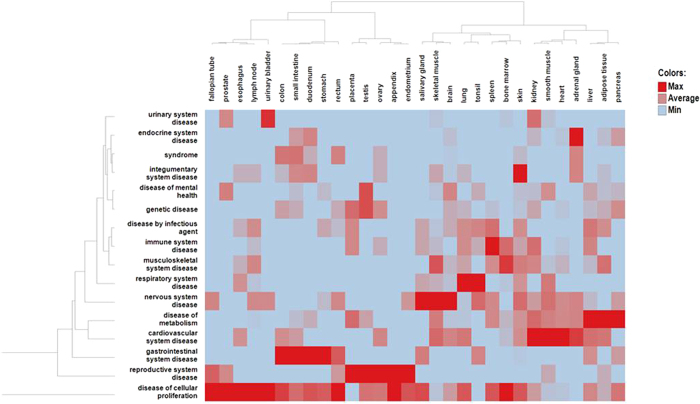
Ward’s hierarchical clustering of all drug-targets and their top three predicted tissue assignments across different disease classes. Each data point represents the number of targets for a specific tissue in a particular disease class. For example, targets associated with *diseases of cellular proliferation* (Cancer) are clustered across several tissues because they are indicated for cancers involving multiple tissues. Similarly *diseases of metabolism* include targets for diseases such as Obesity, Hyperuricemia, and Amylodosis and are often associated with multiple tissues. In contrast, *urinary system diseases* are highly specific in that they target kidney, urinary bladder and prostate. The color scale shown in the figure used only min/max/average values (min = 0, avg = 3.75, max = 28).

Next, we evaluated the tissue distribution of the expressed drug targets with respect to different therapeutic areas as defined by their *Disease Ontology* (Fig. 2). As expected, several therapeutic areas show a similar enrichment for targets expressed in specific tissues, especially *cardiovascular system, nervous system*, and *metabolic diseases*. The exception here are *diseases of cellular proliferation* (i.e. cancers), which are enriched for targets spanning most tissues surveyed. Correspondingly, many drugs for expressed targets are indicated for the treatment of diseases in multiple therapeutic areas (as in Table 2). For example, 23 and 12 targets for *nervous system diseases* are shared with *cardiovascular system disease* and *disease of metabolism* respectively (listed in Table 3). This is consistent with previous observations that both nervous system and metabolic syndrome play important roles in the regulation of cardiovascular function over multiple time scales^11^,^12^. For instance impairment in the sympathetic nervous system (SNS) signaling is one of the common factors implicated in the diabetic heart failure^13^. Glucose, insulin, and free fatty acids produce elevated circulating levels of norepinephrine which contribute to increased sympathetic nervous activity, eventually resulting in enhanced Ca^2+^ influx and cardiac contractility^14^,^15^,^16^. We also found 17 shared targets between *cardiovascular system disease* and *disease of metaboli*sm (listed in Table 3) which may be attributed to the cross-talk between these two systems as well as their common overlap with *nervous system diseases*^17^,^18^.

We then focused on characterizing the remaining 168 (13%) of the gene-disease pairs without detected mRNA or protein expression in the predicted tissue. First, we confirmed that the majority (86%) of the 66 genes represented among these 168 pairs do show a tissue specific pattern of expression. 39 genes are predominantly expressed in a single tissue with at least five-fold higher expression levels in a single tissue compared to average of all other tissues. Another 14 genes have at least five-fold higher levels of expression in up to seven tissues. Of the remaining 13 genes, 4 are expressed without notable tissue enrichment, while only 2 genes (MPL and TRHR) were not detected in any of the 32 tissues in Uhlen *et al*. or the 45 tissues in GTEx, showing an average expression of 0.66 and 0.52 FPKM, respectively, using the tissue with highest expression for each gene.

**Figure 3:**
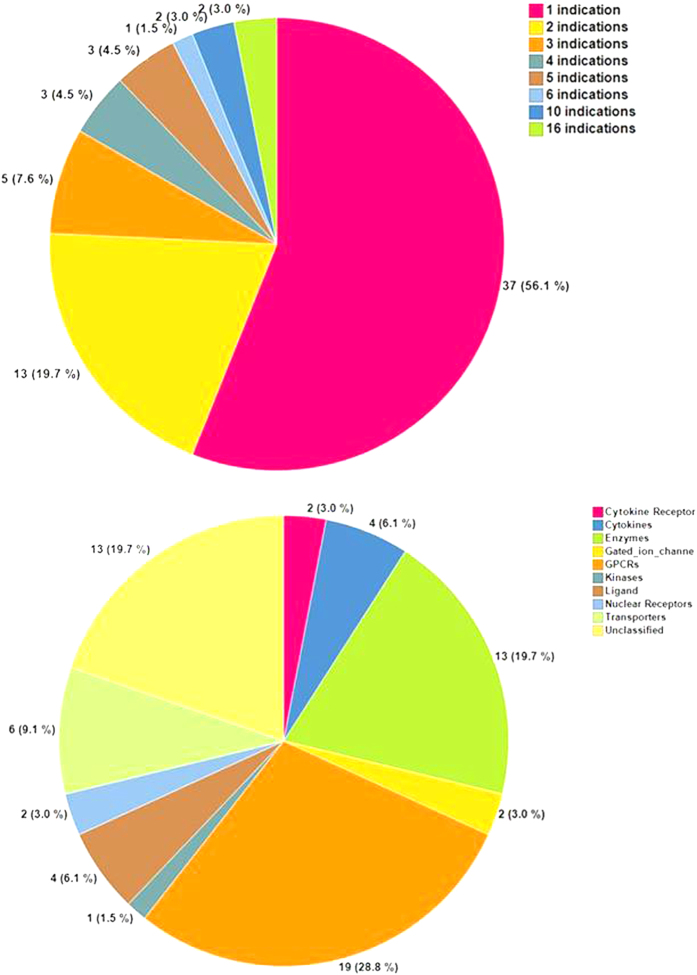
Analysis of the 66 targets whose expression could not be confirmed in an available disease relevant tissue type. (**a**) The number of total indications for each target (**b**). The target classes (protein families) of the 66 drug targets.

Next, we also looked at the distribution of the 168 unconfirmed gene-disease pairs across therapeutic areas. Interestingly, we found that relatively few targets are indicated for multiple indications. 85 (52%) of the unconfirmed gene-disease pairs represent just 11 genes (17%) that each have at least four indications: SLC6A4 (16), IL2RA(16), PTGER1(10), GNRHR(10), SLC6A2(6), SERPINC1(5), OPRM1(5), F2(5), CUBN(4), IL(5) and PGR(4) (Fig. 3a and Supplementary Table I). Further, 41 unconfirmed gene-disease pairs represent another 18 genes with 2 or 3 disease indications. Combined, this shows that drugs with multiple indications (77%, n = 126) are enriched among the unconfirmed gene-disease pairs (P = 3e^−6^, chi-square frequency test).

Given the observations above that most unconfirmed genes both show tissue-specific expression and are targeted for multiple indications, we next asked whether the 66 unconfirmed genes are expressed in a different tissue specified by one of the alternate indications. Indeed, 38 of the 66 (58%) unconfirmed genes are expressed in a different tissue (Fig. 3a). For example, Plasminogen (PLG) is a target indicated for multiple diseases: Conjunctivitis Allergic, Myocardial Infarction, Pulmonary Embolism and Venous Thrombosis. This gene is detected in tissues assigned to 3 of the 4 disease indications with Myocardial Infarction being the only exception. These 38 genes also tend to have robust expression with a median FPKM of 4.8 and detected expression (FPKM > 1) in 10 tissues on average. In contrast, an additional 22 of the 66 genes are associated with a single indication and tend to have weak expression, with a median FPKM of 0.2 and detected expression in 4 tissues on average. These results indicate that the majority of unconfirmed genes are targets of drugs for multiple indications and are expressed in the predicted tissue for at least one other indication.

Another explanation for the 168 unconfirmed gene-disease pairs is that certain classes of drug targets simply may be difficult to measure, due to extreme tissue specificity or localization. In such cases, measured expression may be diluted or absent due to cell-type heterogeneity within a sampled tissue (e.g., brain) or due to absence of a tissue in a given data set. In fact, we identified 30 pairs that were assigned brain as the predicted disease-relevant tissue with high confidence (average MAS score greater than 70%), yet have undetected expression levels in brain. To evaluate these genes further, we employed an independent large-scale mRNA data set (GTEx)^19^ which contains an extensive panel of 13 sampled brain regions. Overall, 6 of the 30 unconfirmed pairs for brain are expressed in one or more of the brain regions in GTEx (3 in pituitary, 3 in nucleus accumbens) (Supplementary Table I). We also confirmed expression for another 5 pairs using GTEx: (2 in salivary gland, 1 apiece in lung, stomach and 1 adrenal gland). These results are consistent with the possibility that some genes remain undetected simply due to current technical limitations in available data, due to tissue sampling bias or resolution, tissue-specific differences in absolute mRNA copy number, or short protein half-life.

Finally, we examined the functional properties of the 66 unconfirmed genes to identify enriched classes (Fig. 3b). The most enriched target classes are G-protein coupled receptors (19 members), enzymes (13), transporters (6), cytokines (4), and unclassified (13) (Supplementary Table I). Notably, 6 of the undetected enzymes are coagulation proteins involved in hemostasis; these genes are expressed in the liver (Supplementary Table I; F2, F9, F10, PLG, PROC, XDH), but were not detected in the tissue predicted to associate best with the disease phenotype. We used DAVID to evaluate shared functional properties by gene ontology enrichment^20^. Notably, 21 genes are involved in cell-cell signaling (GO:0007267, FDR p-value: 2.8e^−12^), 12 of which are directly involved in impairing synaptic transmission (GO:0007268, FDR p-value: 1.5e^−7^) and consist of primarily calcium-channels, GABA transporters, dopamine and serotonin receptors^21^. Many of these drug targets are associated with psychiatric and neurologic disorders, ranging from addiction, mental retardation and autism.

The next most represented class of lowly expressed genes (19) is involved in homeostatic processes (GO: 0042592, FDR p-value: 6e^−7^). Many (10) of these proteins play a key role in calcium ion homeostasis, Ca(^2+^) signaling by regulating multiple neuronal functions, including synaptic transmission, plasticity, and cell survival^22^. We also identified fourteen lowly expressed genes that are central to wound healing response (FDR E-Value: 3.9 e^−5^). Several of them are known regulators of apoptosis and are expressed at high levels when subjected to inflammatory response. Amongst them are seven proteins that are involved in coagulation cascade, a major aspect of wound healing. Notably these genes are selectively expressed only in the liver, but not in tissues associated with cardiovascular diseases such as heart, adipose tissue etc.

## Discussion

In the current study, we evaluated both gene and protein expression variation for clinically successful targets (defined as Phase III or marketed) across a diverse set of normal human tissue types that are relevant to the disease phenotype. By integrating protein and mRNA expression data, we are able to show that a majority (87%) of marketed and Phase III drug targets are expressed at detectable levels in a tissue relevant to the disease under normal conditions. This systematic evaluation reiterates the importance of confirming expression of a target protein in a disease relevant tissue and lends support to previous findings that for a drug treating it to have an effect, its target should be expressed in that tissue under normal conditions. This finding corresponds with a quantitative view of disease progression, whereby genes that are already expressed in normal tissue become more highly or more weakly expressed in the disease state; in contrast to a model in which successful drug targets become selectively expressed only in the diseased state.

Several factors account for the drug target-disease pairs (13%) not detected in the expected tissues. Our analysis of the 66 implicated genes revealed that 86% have a tissue-specific pattern of expression. In fact only 2 of the 345 (2.6%) genes examined did not have detectable expression in any tissue surveyed in two of the largest expression resources provided by Uhlen *et al*. and the GTEx Consortium. Furthermore, 58% of the 66 genes are expressed in to at least one tissue linked to an indication for the drug with multiple indications. Therefore, our inability to detect expression in relevant tissues for all indications is not due to a true absence of mRNA or protein.

Instead, inability to detect tissue-specific expression more likely reflects error (a high false negative rate) either due to limitations in the technology used to detect weakly expressed genes or due to the computational inference of disease-tissue association. Second, the current analysis is limited by the number of tissues for which expression data is available (32 tissues) and therefore the predicted tissue assignments for some of the diseases will be sub-optimal at best. For example, genes associated with breast neoplasms are more likely to be associated with breast. However, as no expression profiles are available in the database from breast tissue per se, our methodology assigned lymph nodes as the most optimal tissue for this disease. Another key limitation is tissue heterogeneity, arising from multiple cell types. A major confounding factor in experiments when applying these genome-wide gene expression profiling methods at whole tissue levels is that the contribution of a specific cell type to the total amount of measured gene expression cannot be determined. As genome scale data from single cell experiments becomes more widely available for multiple tissues, one can extend these data to estimate the extent of cell-type heterogeneity present in existing “bulk tissue” resources, such as GTEx.

Furthermore, inability to detect tissue-specific expression may reflect a true biological phenomenon. For example, a protein may be active or stable even when the mRNA levels are very low. Beyond established differences between mRNA and protein levels for the same gene, there may be proteins which perform their function in relatively low levels. Indeed, we found that several of these genes that are lowly expressed in their disease assigned tissue also appear to have low overall expression levels across all tissues. This suggests that these proteins (or genes) might still be functional in the diseased tissue. Most of the lowly expressed targets are primarily involved in ion transport, regulation of cell apoptosis, and synaptic transmission. One possibility may be that these proteins may not be active in the normal tissues as manifested by their low expression levels, but may be altered in disease state. For example, expression levels of solute carriers (SLCs) are generally higher in a disease states as they are modulated by cytokines, hormones, and growth factors, extracellular signals in response to the metabolic state of the cell affected by stress or other stimuli. Recent advances in quantifying half-lives, transcription and translation rate constants across the entire genome have shown that many fast responding genes have short protein and/or mRNA half-lives^23^. Half-life measurements of proteins and mRNAs can be used to search for properties that characterize these unstable mRNAs and proteins. The challenge, however, is that knowledge of relationships between half-life of mRNA and protein is limited to a few lower organisms and some mammalian cell lines, therefore its applicability to whole organism remains unclear.

The complexity and the dependencies between tissues in a multi-tissue organism may also explain inconsistencies between tissue-specific expression of a target and the locus of disease. For example, most coagulation proteins are expressed in the liver, but are targets for cardiovascular diseases where the primary tissue is heart. Another example is in cancers where a mutation in a protein active in one tissue may result in clinical pathology in different tissue^9^. In summary, the role of weakly expressed genes in causing disease in a specific tissue is complex and will require additional analysis.

## Conclusion

In the current study, we evaluated both tissue-specific gene and protein expression variation for clinically successful targets (defined as targets of Phase III or marketed drugs) across a diverse set of normal human tissue types that are relevant to the disease phenotype. The results of our systematic study broadly agree with previous case studies and reiterate the importance of confirming target expression in normal or healthy state of a disease relevant tissue. Secondly our results highlight the value of comprehensive expression resources that advance expression technology, understanding tissue diversity, and our ability to dissect tissue heterogeneity through single cell technologies. The benchmarking of our methodology on efficacious drug targets shows that normal tissue expression can be used routinely by all drug discoverers when choosing a new drug target to pursue for a particular disease indication. Finally this effort of quantification using measurements taken from mRNA and protein levels should be considered complementary because both these molecular populations are necessary for a complete understanding of the cell behaviour under normal conditions.

In conclusion, our results suggest that in addition tothe currently accepted use of dysregulated genes and pathway as a means of selecting a new drug target, we provide an additional criterion in evaluating potential novel drug targets that involves confirmation of gene expression at the mRNA and protein levels in the proper disease-relevant tissue.

## Methods

### Drug Targets

To benchmark our approach, we compiled a gold standard set of gene-disease relationships by extracting all genes and their disease indications that are currently in Phase III or marketed as per the drug industry pipeline database Pharmaprojects (http://www.citeline.com/products/pharmaprojects/). We restricted ourselves to Phase III and Marketed drugs as for these there is human clinical evidence pointing to efficacy based on the phase II trials. We primarily focused on drugs that listed only a single human protein as their target to avoid the ambiguity associated with multiple targets^24^. We also excluded non-human drug targets, thus excluding most anti-infective targets. We also mapped diseases to MeSH. An additional filtering step removed genes that lacked organ specificity targeting non-specific disease terms like Inflammation and Neoplasms. The final list produced a set of 1,305 unique disease-gene associations spanning 345 targets and 406 diseases (Supplementary Table S1). In addition, each MeSH disease term was mapped to the Human Disease Ontology (DO) (http://www.disease-ontology.org). DO is a biomedical resource of standardized common and rare disease concepts with stable identifiers organized by disease etiology^25^.

Target class assignments for each drug target was applied according to the molecular function of the gene product using the Swissprot accession numbers^26^. The breakdown of the 345 drug targets is: 69 enzymes, 68 G-protein coupled receptors (GPCRS), 27 transporters, 15 cytokine receptors, 12 cytokines, 11 nuclear receptors, 11 kinases, 10 ligands, 7 structural and adhesion proteins, 5 ligand-gated ion channel receptors and 4 others. The remaining 94 targets could not be assigned a target class and were designated as “Unclassified”.

### RNA-Seq Expression

Tissue-specific gene expression data set expression profiles for 32 tissues/organs based on RNA-Seq analyses of 122 individual samples, including classification of tissue specificity and predictions of secreted and transmembrane regions was downloaded from the supplementary section of recent publication by Uhlen^8^. Quantification scores for each gene/transcripts across all 122 samples are represented in FPKM (fragments per kilobase of exon model per million mapped reads) values. A cutoff value of 1 FPKM was used as a limit for detection across all tissues. A total of 20,344 unique genes were measured. The ensuing data was log-transformed and normalized as described by Uhlen^8^.

GTEx v6 RNA-seq data were downloaded from http://gtexportal.org and summarized by computing the median FPKM value for each gene across replicate samples of each tissue. For each of the 163 undetected gene-indication pairs, the median FPKM value is reported for the tissue indicated in the “Disease-Tissue Association” column of Supplementary Table S1. For cases where multiple relevant tissues exist (e.g. multiple brain regions were sampled), the tissue with the highest median FPKM was reported. If a specified tissue was not sampled in GTEx, a suitable proxy was used (e.g. EBV transformed lymphoblasts was used for lymph node; whole blood was used for bone marrow), otherwise NA was reported (e.g. placenta).

### Proteomics Expression

Evidence for the existence of protein was obtained by downloading data from the Human Protein Atlas (HPA)^8^. The current Version 13 of the Human Protein Atlas contains protein data for 83% of the predictive human genes and is derived from 44 different human tissues. The Human Protein Atlas based on immunohistochemistry contains images and protein profiles showing the spatial distribution of proteins in 44 different normal human tissues and 20 different cancer types, as well as 46 different human cell lines based. In total, the new Human Protein Atlas contains expression profiles at the tissue or sub cellular level based on 24,028 antibodies toward 16,975 genes. Each protein is annotated with a “reliability score” to indicate the level of reliability of the analyzed protein expression pattern based on available protein/RNA/gene characterization data. Only genes defined as “supportive”, taking into account only antibodies with HPA evidence of high and medium reliability, according to the latest Human Proteome Project metrics guidelines were considered in this analysis^27^.

### Disease-tissue Associations

The disease-tissue association matrix was generated according to the method defined by Lage *et al*.^27^. The association of a tissue and a disease was estimated by measuring their co-occurrence in PubMed abstracts relative to the number of abstracts mentioning the disease or tissue term alone. The disease search terms was limited to PubMed abstracts in which they were qualified as major MeSH^28^ topics of the article, while the tissue search term was more general limiting to a MeSH topic (no requirement for major). For example, search terms “psoriasis[majr]” and “skin[mh]” was used to measure abstracts that mention psoriasis or skin alone respectively. In order to measure the co-occurrences, the search term “psoriasis[majr] AND skin[mh]” was utilized. The maximal association score (MAS) was computed using the results from the search terms based on the approach using Ochiai’s coefficient^29^, and then normalized by the sum of all OCs for the each disease as shown previously^7^. These scores range between 0 and 100 with 100 representing the most specific tissue for that particular disease. The entire MAS matrix can be found in Supplementary Table S2. For tissue assignments, we picked the top three tissues with highest MAS score provided they met the threshold score of > = 8%. Increasing this number further did not change the number of observed expression trends significantly (Supplementary Table S3). The final step for assigning a single tissue to each gene-disease association was based on picking the tissue that either showed the highest levels of mRNA/gene expression or could be detected at the protein level (Supplementary Table S1).

### Statistical analysis

For each gene disease pair, we created matched pairs of expression in the disease-relevant tissue to average expression all other tissues. We did a Paired *t* test across all the gene-disease pairs to compare the average expression *Z* score over all disease genes in the most disease-relevant tissue to the other tissues. A level of *p* < 0.05 was considered significant.

## Additional Information

**How to cite this article**: Kumar, V. *et al*. Systematic Analysis of Drug Targets Confirms Expression in Disease-Relevant Tissues. *Sci. Rep.* **6**, 36205; doi: 10.1038/srep36205 (2016).

**Publisher’s note:** Springer Nature remains neutral with regard to jurisdictional claims in published maps and institutional affiliations.

## Supplementary Material

Supplementary Information

## Figures and Tables

**Table 1:** A select subset of the unique gene-disease associations with their predicted tissue assignments and gene expression intensities.

Gene	Disease Name	Disease Ontology	Predicted Tissue	MAS Score	mRNA Detected	mRNA Intensity (FPKM)	Protein Detected
*ACPP*	Prostatic Neoplasms	disease of cellular proliferation	prostate	71.1	Y	1916.41	Y
*AGTR1*	Diabetic Nephropathies	urinary system disease	kidney	84.66	Y	11.97	N
*CD22*	Lymphoma, B-Cell	disease of cellular proliferation	lymph node	30.58	Y	267.91	Y
*CHRM1*	Sjogren’s Syndrome	immune system disease	salivary gland	79.04	Y	18.19	N
*DMD*	Muscular Dystrophy, Duchenne	nervous system disease	skeletal muscle	74.91	Y	41.85	Y
*GLA*	Fabry Disease	disease of metabolism	kidney	28.81	Y	14.6	Y
*HCAR2*	Brain Ischemia	cardiovascular system disease	brain	85.26	N	0.12	Y
*MC2R*	Addison Disease	endocrine system disease	adrenal gland	59.95	Y	36.71	N
*PPIA*	Arthritis, Rheumatoid	musculoskeletal system disease	lymph node	10.27	Y	308.36	Y
*PRKCD*	Keratosis, Actinic	disease of cellular proliferation	skin	100	Y	20.55	N
*SCN9A*	Neuralgia	nervous system disease	brain	33.83	N	0.68	Y
*SLC12A3*	Hypertension	cardiovascular system disease	kidney	33.3	Y	142.87	Y
*TUBB*	Pancreatic Neoplasms	disease of cellular proliferation	pancreas	49.23	Y	55.13	Y
VDR	Keratosis	integumentary system disease	skin	81.82	Y	15.85	N

The entire list for all 1,305 unique gene-disease associations spanning 345 targets and 406 diseases can be found as Supplementary Table S1.

**Table 2:** Number of Drug-Targets shared between disease classes.

Disease Class	Cardiovascular system Disease	Disease by infectious agent	Disease of cellular proliferation	Disease of mental health	Disease of metabolism	Endocrine system disease	Gastrointestinal system disease	Genetic disease	Immune system disease	Integumentary system disease	Musculoskeletal system disease	Nervous system disease	Reproductive system disease	Respiratory system disease	syndrome	Thoracic disease	Urinary system disease
Cardiovascular system disease	70																
Disease by infectious agent	4	20															
Disease of cellular proliferation	12	8	88														
Disease of mental health	8	2	5	29													
Disease of metabolism	17	2	8	8	66												
Endocrine system disease	1	2	2	1	5	9											
Gastrointestinal system disease	6	7	7	3	6	5	41										
Genetic disease	5	0	4	3	7	1	3	18									
Immune system disease	12	6	12	3	6	3	8	4	45								
Integumentary system disease	8	5	9	5	7	4	11	4	8	47							
Musculoskeletal disease system	11	5	9	3	8	6	7	4	9	14	39						
Nervous system disease	23	6	14	15	12	2	11	6	11	18	15	90					
Reproductive system disease	5	3	7	7	5	2	6	1	4	8	4	9	19				
Respiratory system disease	8	4	5	2	1	2	7	2	8	9	5	9	4	24			
Syndrome	4	2	1	1	1	1	8	0	2	6	4	7	2	1	19		
Thoracic disease	0	0	1	1	0	0	0	0	0	0	0	0	1	0	0	1	
Urinary system disease	11	4	5	2	7	3	4	2	5	4	4	8	3	4	1	0	18

**Table 3:** List of targets shared between two different disease classes.

Disease Ontology 1	Disease Ontology 2	Shared Targets	Targets
*nervous system disease*	*cardiovascular system disease*	23	ADRA1A;ADRA2A;ADRB1;AVPR2;CA2;CACNA1C;HMGCR;HTR2A;IL1B;INSR;MS4A1;MTOR;NR3C1;PDE5A;PLG;PPARG;PPIA;PTGER1;SLC12A3;SLC6A4;TBXAS1;TNF;VEGFA;
*nervous system disease*	*disease of metabolism*	12	AKR1B1;AVPR2;CNR1;CUBN;DRD2;HMGCR;IL1B;INSR;NR3C1;PGR;PPARG;SLC6A4;
*disease of metabolism*	*cardiovascular system disease*	17	ACE;AGTR1;AVPR2;CETP;HCAR2;HMGCR;IL1B;INSR;LPL;NR3C1;PDE3A;PPARG;SLC22A12;SLC5A2;SLC6A2;SLC6A4;XDH;

The complete list of all shared targets between all disease classes can be found in Supplementary Table S4.
